# Efficacy and safety of disitamab vedotin (RC48) combined with camrelizumab and S‐1 for neoadjuvant therapy of locally advanced gastric cancer with HER2‐overexpressing: Preliminary results of a prospective, single‐arm, phase II study

**DOI:** 10.1002/ctm2.70679

**Published:** 2026-05-06

**Authors:** Longgang Wang, Bing Liu, Luguang Liu, Liqing Liu, Rong Li, Shumei Han, Xu Sun, Bo Bi, Yuanlin Sun, Dong Sun, Jie Chai

**Affiliations:** ^1^ Department of Gastrointestinal Surgery Shandong Cancer Hospital and Institute, Shandong First Medical University and Shandong Academy of Medical Sciences Jinan China; ^2^ Gastric Surgery Ward Shandong Cancer Hospital and Institute, Shandong First Medical University and Shandong Academy of Medical Sciences Jinan China; ^3^ Department of Gastroenterology Shandong Cancer Hospital and Institute, Shandong First Medical University and Shandong Academy of Medical Sciences Jinan China

**Keywords:** antibody‒drug conjugate, disitamab vedotin, G/GEJ cancer, HER2‐overexpressing, neoadjuvant therapy

## Abstract

**Purpose:**

Perioperative treatment of gastric and gastroesophageal junction (G/GEJ) cancer is evolving towards multimodal strategies incorporating HER2‐targeted therapy, immunotherapy and chemotherapy. Disitamab vedotin (RC48), an HER2‐targeted antibody–drug conjugate, shows promising antitumour activity and potential synergy with immune checkpoint inhibitors. This study evaluated neoadjuvant RC48 combined with camrelizumab and S‐1 in resectable HER2‐overexpressing locally advanced G/GEJ adenocarcinoma.

**Methods:**

Patients with histologically confirmed HER2‐overexpressing (IHC 3+ or 2+) resectable G/GEJ cancer staged as cT3‐4aN1‐3M0 were enrolled in this prospective single‐arm phase II study. Patients received three 3‐weekly cycles of RC48, camrelizumab and S‐1 before surgery. Pathological complete response (pCR) was defined as the primary endpoint, whereas major pathological response (MPR), objective response rate (ORR), tumour downstaging, disease‐free survival (DFS), overall survival (OS) and safety were evaluated as secondary endpoints. Exploratory circulating tumour DNA (ctDNA) methylation profiling (PredicineEPIC) assessed molecular response dynamics and ERBB2 copy number variation.

**Results:**

From 18 September 2022 to 12 December 2024, 32 patients were enrolled; 24 proceeded to D2 resection. The ORR after neoadjuvant therapy was 80.0% (24/30). In the surgical cohort, pCR and MPR were achieved in 25.0% (6/24) and 45.8% (11/24) of patients, respectively, with an R0 resection rate of 100%. The median DFS and OS were not reached at the time of analysis. In the ctDNA substudy (*n* = 14), methylation‐derived tumour fraction declined during therapy and ERBB2 plasma copy number gain aligned with tissue HER2 status. Treatment‐related adverse events of grade ≥3 were reported in 31.3% of patients.

**Conclusion:**

Neoadjuvant RC48 combined with camrelizumab and S‐1 showed potential antitumour activity with an acceptable safety profile in HER2‐overexpressing locally advanced resectable G/GEJ adenocarcinoma.

**Key points:**

- Neoadjuvant RC48 plus camrelizumab and S‐1 showed encouraging pathological responses in HER2‐overexpressing gastric cancer.
- The regimen achieved a pCR rate of 25% and an MPR rate of 45.8%.
- The combination therapy demonstrated a manageable safety profile.
- Exploratory ctDNA methylation analysis suggested potential for dynamic response monitoring.

## Graphical abstract

Neoadjuvant disitamab vedotin combined with camrelizumab and S‐1 achieved high pathological response rates with manageable safety in resectable HER2‐overexpressing gastric and gastroesophageal junction cancer. Longitudinal circulating tumour DNA methylation profiling enabled dynamic monitoring of molecular response during treatment.

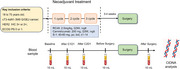

## 1 INTRODUCTION

Gastric cancer (GC) is a biologically heterogeneous malignancy with aggressive clinical behaviour and a particularly high burden in East Asia.^1^ According to the latest statistical data, GC ranks fifth in both incidence and mortality among all malignancies.^2^, ^3^ Early‐stage GC often presents with non‐specific symptoms and is associated with low public awareness regarding the importance of early detection, which complicates timely diagnosis.^4^ Consequently, approximately half of the patients are diagnosed at stages III or IV, posing significant therapeutic challenges. Perioperative therapy combined with D2 gastrectomy is the standard of care for locally advanced disease.^5^, ^6^, ^7^ Neoadjuvant therapy has been shown to effectively downstage tumours, reduce tumour burden, increase the likelihood of R0 resection, and lower the risks of recurrence and metastasis, ultimately contributing to improved patient outcomes.^8^, ^9^ Despite these benefits, the overall improvement following neoadjuvant chemotherapy remains inadequate.^10^, ^11^ Therefore, optimising neoadjuvant strategies to enhance pathological response and long‐term survival in patients with locally advanced gastric or gastroesophageal junction (G/GEJ) cancer remains an important area of ongoing research. In China, neoadjuvant treatment for locally advanced GC is commonly based on two‐drug chemotherapy regimens combining fluoropyrimidines and platinum agents.^9^, ^12^ Although these regimens have been associated with improved resection rates and survival, they remain insufficient in terms of pathological complete response (pCR) rates and long‐term disease control. Additionally, chemotherapy‐associated toxicities often limit treatment tolerance, particularly in elderly or frail patients. These limitations highlight the urgent need for more effective and tolerable neoadjuvant strategies, particularly for patients with HER2‐overexpressing disease.

Targeting HER2 has become an important strategy in the treatment of GC, primarily due to its role in promoting downstream signaling pathways that stimulate the growth and survival of GC cells.^13^, ^14^ Numerous studies have demonstrated that HER2‐overexpressing is associated with a poor prognosis in patients with operable GC.^15^ In Chinese GC patients, the HER2‐overexpressing rate is approximately 11.5%.^16^ The ToGA trial introduced HER2‐targeted therapy into the management of advanced GC, leading to the adoption of trastuzumab plus chemotherapy as the standard first‐line treatment for patients with HER2‐overexpressing stage IV disease.^17^ The phase III KEYNOTE‐811 trial showed that pembrolizumab added to trastuzumab‐based chemotherapy improved response rates in HER2‐overexpressing advanced GC, supporting combined HER2‐targeted and immunotherapy approaches.^18^ HER2‐targeted antibody–drug conjugates (ADCs), including disitamab vedotin (DV, RC48)^19^ and trastuzumab deruxtecan,^20^ have emerged as novel agents that deliver cytotoxic payloads to HER2‐expressing tumour cells. However, their efficacy as monotherapy in the neoadjuvant setting appears limited. For example, the multicentre phase II study EPOC2003 demonstrated that the major pathological response (MPR) rate of neoadjuvant therapy with trastuzumab deruxtecan for HER2‐overexpressing G/GEJC was 14.8%, while the pCR rate was only 3.7%.^21^ Due to limited efficacy of conventional chemotherapy, perioperative anti‐HER2 strategies for locally advanced GC are under active investigation. The NEOHX study reported a pCR rate of 9.6% with trastuzumab plus XELOX, supporting the potential role of anti‐HER2 therapy in this setting.^22^

Emerging data indicate that integrating immune checkpoint inhibitors (ICIs) with chemotherapy can improve therapeutic outcomes in the neoadjuvant or perioperative management of GC. The phase III MATTERHORN trial further showed that adding durvalumab to perioperative FLOT resulted in superior event‐free survival compared with FLOT alone in patients with resectable G/GEJ adenocarcinoma.^23^ Durvalumab was associated with a higher pCR rate (19.2% vs. 7.2%) and an acceptable safety profile, supporting its use in perioperative therapy. However, these findings originate from different clinical trials with varying study designs, patient populations and treatment regimens; therefore, cross‐trial comparisons should be interpreted cautiously.

RC48, an HER2‐directed ADC, delivers the microtubule inhibitor monomethyl auristatin E through a cleavable linker and has demonstrated encouraging antitumour activity with a tolerable safety profile in HER2‐overexpressing GC.^19^, ^24^, ^25^ A recent multicentre phase II study evaluating RC48, tislelizumab and S‐1 as first‐line therapy in HER2‐overexpressing advanced G/GEJ cancer reported an objective response rate (ORR) of 89.4%, a median progression‐free survival (PFS) of 12.7 months and an 18‐month overall survival (OS) rate of 72.7%, suggesting favourable efficacy and tolerable safety.^26^ However, the application of RC48 in combination with ICIs and chemotherapy for perioperative anti‐HER2 therapy in LAGC remains unexplored. Treatment of locally advanced G/GEJ cancer remains challenging, with no established perioperative regimen for HER2‐overexpressing disease.

In this phase II trial, we evaluated the efficacy and safety of neoadjuvant RC48 in combination with camrelizumab and S‐1 for patients with HER2‐overexpressing locally advanced resectable G/GEJ cancer.

## 2 METHODS

### 2.1 Study design

Registered under ChiCTR2300075446, this investigator‐initiated, open‐label study evaluated the safety, tolerability and efficacy of neoadjuvant RC48 in combination with camrelizumab and S‐1 in patients with HER2‐overexpressing locally advanced GC. Ethical approval was obtained from the Institutional Ethics Committee of Shandong Cancer Hospital and Institute, Shandong First Medical University (SDZLEC2022‐152‐02). The study adhered to the principles of Good Clinical Practice and the Declaration of Helsinki, as well as relevant local regulations. Written informed consent was obtained from all participants prior to enrollment.

### 2.2 Patients

Patients were eligible if they met the following criteria: (1) aged 18–75 years; (2) no prior treatment should have been administered; (3) participants must have histologically and/or cytologically confirmed resectable GC/GEJ cancer; (4) clinical stage cT3‐4aN1‐3M0, as determined by endoscopic ultrasound or contrast‐enhanced computed tomography or magnetic resonance imaging, according to the 8th edition of the American Joint Committee on Cancer tumour, node, metastases (TNM) classification^27^; (5) HER2 status assessed by Immunohistochemistry (IHC) as 3+ or 2+ (irrespective of FISH), according to the established scoring system for GC^28^; (6) Eastern Cooperative Oncology Group performance status of 0–1; (7) a tumour sample was required for assessment of programmed cell death protein 1/ programmed cell death ligand (PD‐1/PD‐L1) expression and microsatellite instability (MSI) status. Exclusion criteria included active autoimmune disorders, concurrent malignancies identified at screening, active hepatitis B or C infection, chronic enteritis, or known hypersensitivity or intolerance to the study medications or their components. Patients with human immunodeficiency virus (HIV) infection were not eligible. In addition, pregnancy or lactation, as well as being of reproductive potential without the use of effective contraception, also precluded participation.

### 2.3 Sample size calculation

The efficacy and safety of neoadjuvant RC48 in combination with camrelizumab and S‐1 were evaluated in patients with HER2‐overexpressing G/GEJ cancer. As this was an exploratory study, no formal statistical calculation was applied for sample size determination. Approximately 30 patients were targeted based on evidence from prior neoadjuvant studies in GC. To account for potential dropout during treatment or before surgery, the final enrollment was set at 32 patients.

### 2.4 Procedures

Three cycles of neoadjuvant therapy were administered, including RC48 (2.5 mg/kg, every 3 weeks [Q3W], intravenous infusion), camrelizumab (200 mg, Q3W, intravenous infusion) and S‐1 (40–60 mg twice daily on days 1–14 of each 3‐week cycle). S‐1 dosing was stratified according to body surface area (BSA): 40 mg for BSA <1.25 m^2^, 50 mg for BSA 1.25‒1.5 m^2^, and 60 mg for BSA ≥1.5 m^2^. Upon completion of neoadjuvant therapy, imaging evaluations are performed and patients without tumour progression are scheduled for surgery within 3‒4 weeks. Patients with resectable tumours underwent total or subtotal distal gastrectomy with D2 lymph node dissection. Following this, adjuvant treatment plans are developed by the researchers based on the individual conditions of the patients.

### 2.5 Endpoint

pCR served as the primary endpoint and was defined by the absence of residual tumour cells in both resected specimens and sampled lymph nodes. MPR, corresponding to Becker grade Ia–Ib, was included among the secondary endpoints. Pathological response was evaluated using the Becker tumour regression grading (TRG) system, a commonly adopted method in GC after neoadjuvant therapy that facilitates comparison across studies. Other secondary endpoints comprised (ORR; complete response [CR] and partial response [PR]), disease control rate (DCR; CR, PR and stable disease [SD]), disease‐free survival (DFS), OS, clinical downstaging rate, R0 resection rate and safety outcomes. DFS referred to the time from surgery to disease progression, recurrence or death from any cause, whereas OS was calculated from enrollment to death from any cause.

### 2.6 Analysis populations

The analysis included three predefined populations: full analysis set (FAS), safety analysis set (SAS) and surgery analysis set. Patients were assigned to the FAS if they had received at least one cycle of neoadjuvant therapy and were assessable for efficacy. Those who received at least one dose of study treatment and had available safety data were categorised into the SAS. Patients undergoing radical gastrectomy after neoadjuvant therapy were included in the surgery set. Evaluation of pathological response, including pCR and MPR, was performed primarily within the surgery set.

### 2.7 Exploratory circulating tumour DNA methylation analysis

To investigate the potential of liquid biopsy for dynamic response monitoring, an exploratory analysis of plasma‐derived circulating tumour DNA (ctDNA) was performed in a subset of patients (*n* = 14) at multiple timepoints during the neoadjuvant period. Whole‐genome methylation profiling was conducted using the PredicineEPIC assay, a cfDNA‐based, ultra‐sensitive next‐generation sequencing platform optimised for low‐input samples.

Tumour fraction (TFx) was estimated based on methylation signal patterns, enabling non‐invasive monitoring of tumour burden. Differentially methylated fragments (DMFs) located in gene promoter regions were identified and subsequently annotated to their corresponding genes. These promoter‐associated DMFs were visualised using heatmap clustering to illustrate methylation patterns across samples. Gene Ontology (GO) enrichment analysis was then performed based on the genes annotated from promoter‐associated DMFs to explore biological processes potentially associated with these methylation changes.^29^ ERBB2 copy number variation (CNV) was also assessed in plasma samples to evaluate concordance with tissue HER2 status.

### 2.8 Statistical analysis

Counts and percentages were used to summarise categorical variables, whereas continuous data are presented as medians with interquartile ranges or ranges. Confidence intervals (95% CIs) for pCR, MPR, R0 resection rate, ORR and DCR were obtained using the Clopper–Pearson method. Kaplan–Meier estimates were applied to evaluate DFS, OS and median follow‐up. Statistical significance was determined using two‐sided tests with a threshold of *p* < .05. All analyses were performed in SPSS Statistics version 25.0.

## 3 RESULTS

### 3.1 Patient characteristics

**FIGURE 1:**
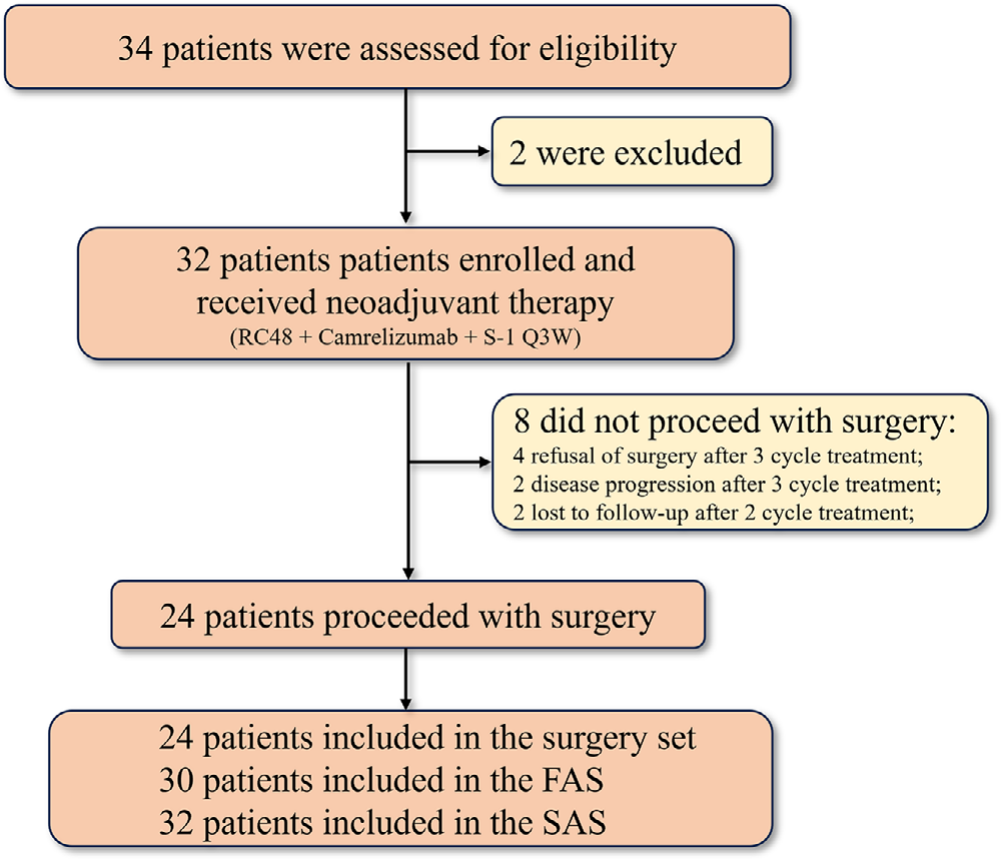
Consort diagram. FAS: full analysis set; Q3W: every 3 weeks; RC48: disitamab vedotin; SAS: safety analysis set.

Between 18 September 2022 and 12 December 2024, a total of 34 patients were screened for eligibility, with two patients excluded from enrollment in this study. Consequently, 32 patients were enrolled and received the treatment, thereby qualifying for inclusion in analysis set (Figure 1). Additionally, eight patients did not proceed to surgery due to refusal of surgery (*n* = 4, three cycles of treatment), disease progression (*n* = 2, three cycles of treatment) and loss to follow‐up (*n* = 2, two cycles of treatment). A total of 24 patients underwent surgery and were included in the surgical cohort. Among all 32 patients, the median age was 65 years (range: 34–78), and males accounted for the majority (30/32, 93.8%). Primary tumours were most commonly located in the stomach (18/32, 56.3%), and all patients were classified as clinical stage III (Table 1). A cT4a stage was identified in 18 patients (56.3%), and nodal involvement (N1–N3) was present in all patients (32/32, 100%). Regarding immunohistochemical baseline characteristics, 24 patients (75.0%) exhibited microsatellite stability/proficient mismatch repair (MSS/pMMR), and 15 patients (46.9%) were classified as HER2 3+. Furthermore, 11 patients (34.4%) were evaluated for PD‐L1 combined positive score (CPS) ≥1 expression.

**TABLE 1:** Baseline characteristics.

Characteristics	All patients (*n* = 32)
Age (years), median (range)	65 (34‒78)
Sex	
Male	30 (93.8%)
Female	2 (6.2%)
Primary tumour location, *n* (%)	
Gastric	18 (56.3%)
Gastroesophageal junction	14 (43.7%)
Clinical T stage, *n* (%)	
cT3	14 (43.7%)
cT4a	18 (56.3%)
Clinical N stage, *n* (%)	
cN1	14 (43.8%)
cN2	14 (43.8%)
cN3	4 (12.5%)
Histologic grade, *n* (%)	
G2	17 (53.1%)
G3	9 (28.1%)
Not evaluable	6 (18.8%)
MSI status, *n* (%)	
MSI‐H/dMMR	1 (3.1%)
MSS/pMMR	24 (75.0%)
Not reported or invalid	7 (21.9%)
HER2 status, *n* (%)	
2+	16 (50.0%)
3+	16 (50.0%)
PD‐L1 CPS, *n* (%)	
CPS < 1	10 (31.3%)
CPS ≥ 1	11 (34.4%)
Not reported or invalid	11 (34.4%)
Clinical tumour, node, metastases stage, *n* (%)	
III	32 (100.0%)
ECOG PS, *n* (%)	
0	24 (75.0%)
1	8 (25.0%)

Abbreviations: ECOG PS, Eastern Cooperative Oncology Group performance status; MSI, microsatellite instability; MSI‐H/dMMR, microsatellite instability‐high/deficient mismatch repair; MSS/pMMR, microsatellite stability/proficient mismatch repair.

### 3.2 Pathological responses

**FIGURE 2:**
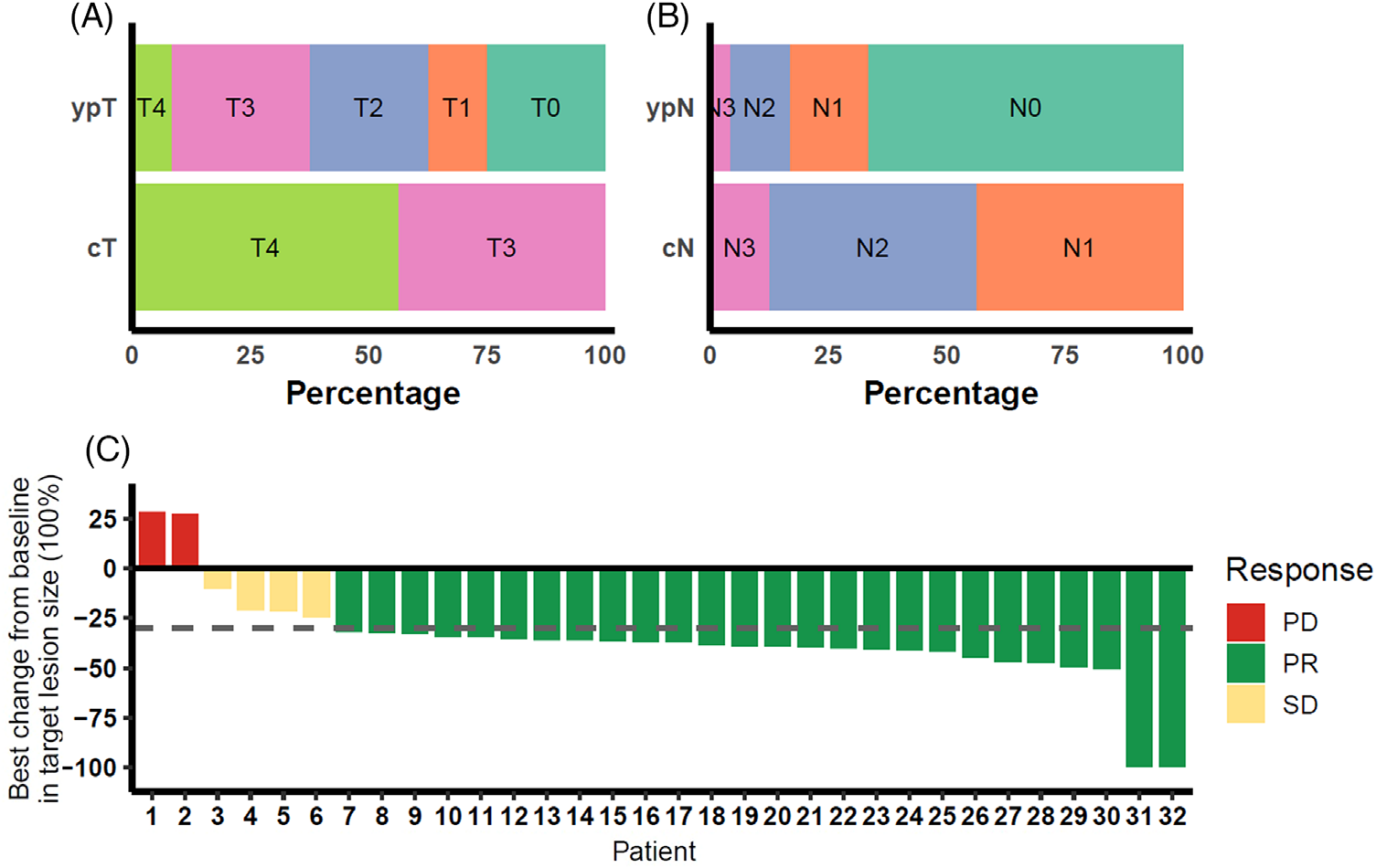
Pathological response in surgery set and tumour response in surgery and full analysis set (FAS) set. (A) Distribution of clinical T stage before treatment (cT) and pathological T stage after surgery (ypT) in the surgery set. (B) Distribution of clinical N stage before treatment (cN) and pathological N stage after surgery (ypN) in the surgery set. (C) Waterfall plot showing the best percentage change from baseline in the sum of target lesion diameters in the FAS according to RECIST 1.1 criteria. Each bar represents an individual patient. Colours indicate the best overall response (progressive disease [PD], partial response [PR] or stable disease [SD]).

A total of 24 patients proceeded to surgery after neoadjuvant therapy (surgery set, *n* = 24). Total gastrectomy with D2 lymphadenectomy was performed in all cases, yielding an R0 resection rate of 100%. pCR was occurred in six cases (25.0%; 95% CI: 12.0%–44.9%). TRG1b was observed in five patients (20.8%), while TRG2 and TRG3 were noted in 10 patients (41.7%) and three patients (12.5%), respectively. The MPR (Becker TRG1a/1b) rate was 45.8% (95% CI: 27.9%–64.9%). In an intention‐to‐treat analysis including all enrolled patients (*n* = 32), patients who did not undergo surgery were considered non‐responders, resulting in a pCR rate of 18.8% (6/32) and an MPR rate of 34.4% (11/32). The pathological T stages after surgery were ypT0 in six patients (25.0%), ypT1 in three patients (12.5%), ypT2 in six patients (25%), ypT3 in seven patients (29.2%) and ypT4a in two patients (8.3%). Based on the pathological N stage after surgery, 16 patients (66.7%) were classified as ypN0, four patients (16.7%) as ypN1, three patients (12.5%) as ypN2 and one patient (4.2%) as ypN3 (Table 2 and Figure 2A,B). Notably, among patients with baseline nodal involvement, 66.7% achieved nodal downstaging to ypN0, indicating a substantial reduction in nodal disease burden with this neoadjuvant regimen.

**TABLE 2:** Pathological response in the surgery set.

Pathological response	Total (*n* = 24)
R0 resection	100%
TRG 1a	6 (25.0%)
TRG 1b	5 (20.8%)
TRG 2	10 (41.7%)
TRG 3	3 (12.5%)
pCR	6 (25%, 95% CI: 12.0%‒44.9%)
MPR	11 (45.8%, 95% CI: 27.9%‒64.9%)
ypT0	6 (25%)
ypT1b	3 (12.5%)
ypT2	6 (25%)
ypT3	7 (29.2%)
ypT4	2 (8.3%)
ypN0	16 (66.7%)
ypN1	4 (16.7%)
ypN2	3 (12.5%)
ypN3	1 (4.2%)

Abbreviations: CI, confidence interval; MPR, major pathological response; pCR, pathological complete response.

### 3.3 Tumour responses

Among the 32 patients included in the FAS, 30 patients (93.8%) completed three cycles of treatment. However, eight patients declined surgery. The majority of patients experienced tumour shrinkage, with over two‐thirds achieving PR, while a few showed SD or progression (Figure 2C). Based on the preoperative imaging assessment, an ORR of 80.0% (24/30, 95% CI: 62.7%‒90.5%) was recorded, which included three patients (10.0%) achieving CR and 21 patients (70.0%) achieving PR according to RECIST v1.1 criteria. Additionally, the DCR was reported in 28 patients (93.33%, 95% CI: 78.7%‒98.2%), with four patients (13.33%) exhibiting SD and two patients (6.67%) demonstrating progressive disease prior to surgery (Table 3).

**TABLE 3:** Radiological response in the full analysis set (FAS) set.

Best responses	Total (*n* = 30)
Complete response	3 (10%)
Partial response	21 (70.0%)
Stable disease	4 (13.33%)
Progressive disease	2 (6.67%)
ORR	24 (80.0%, 95% CI: 62.7%‒90.5%)
DCR	28 (93.3%, 95% CI: 78.7%‒98.2%)

Abbreviations: CI, confidence interval; DCR, disease control rate; ORR, objective response rate.

### 3.4 Tumour markers

**FIGURE 3:**
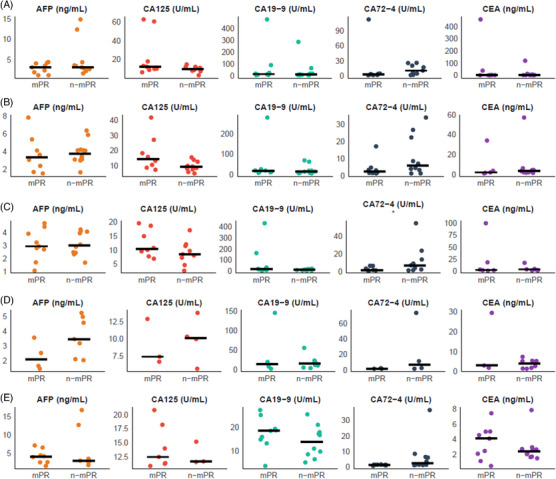
Tumour markers of carcinoembryonic antigen (CEA), alpha‐fetoprotein (AFP), CEA125, cancer antigen (CA)19‐9 and CA72‐4 before and after treatment. (A) The tumour markers of baseline. (B) The tumour markers after C1D1. (C) The tumour markers after C2D1. (D) The tumour markers after C3D1. (E) The tumour markers after surgery (^*^p <  .05).

Serum tumour markers including carcinoembryonic antigen (CEA), alpha‐fetoprotein (AFP), cancer antigen (CA)125, CA19‐9 and CA72‐4 were monitored during the neoadjuvant treatment period (Figure 3A‒E). The tumour markers in patients who achieved MPR were consistent with those in non‐MPR patients at baseline (*p *> .05). Overall, modest decreases in several tumour markers were observed after treatment compared with baseline, with a numerically greater reduction in patients achieving MPR. Among these markers, CA72‐4 showed a significant decline after C1D1 (*p* < .05).

**FIGURE 4:**
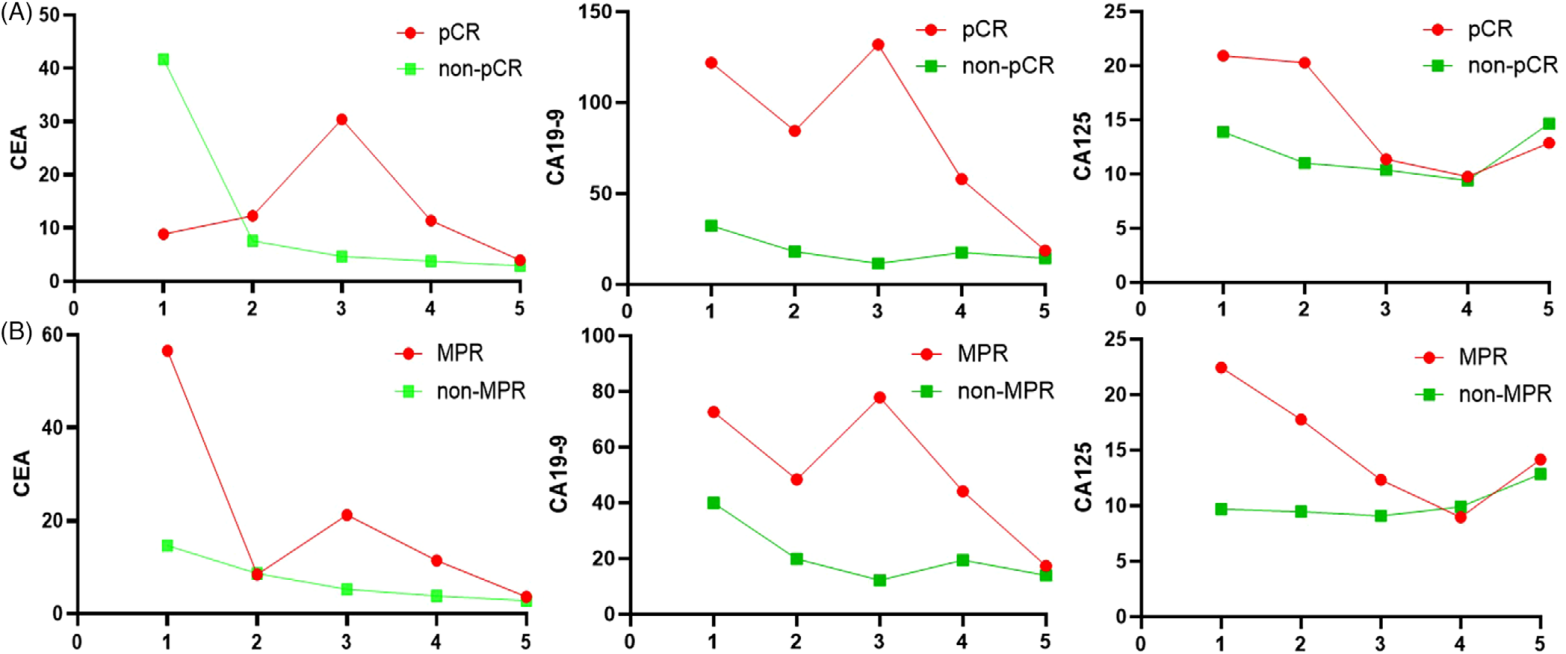
Dynamic changes of tumour markers during perioperative treatment. (A) Serial changes in serum tumour markers (carcinoembryonic antigen [CEA], cancer antigen [CA]19‐9 and CA125) at five timepoints (baseline, C1D1, C2D1, C3D1 and after surgery) in patients with pathological complete response (pCR) and non‐pCR. (B) Serial changes in serum tumour markers (CEA, CA19‐9 and CA125) in patients with major pathological response (MPR) and non‐MPR. In both panels, the pCR/MPR group is shown in purple, and the non‐pCR/non‐MPR group in blue. Tumour marker levels were generally decreased over time during neoadjuvant treatment and after surgery, with distinct patterns between responders and non‐responders.

To further explore biomarker dynamics, longitudinal changes in CEA, CA19‐9 and CA125 were evaluated across five perioperative timepoints (baseline, C1D1, C2D1, C3D1 and post‐surgery). Tumour marker levels generally declined during treatment in both response groups, although differences between responders (pCR/MPR) and non‐responders were not statistically significant (Figure 4A,B). Given the limited sample size, these findings should be interpreted cautiously.

### 3.5 Exploratory ctDNA methylation dynamics

**FIGURE 5:**
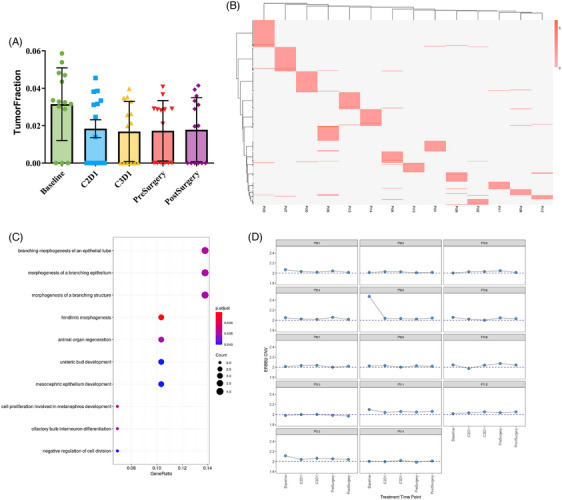
Exploratory analysis of cfDNA methylation dynamics. (A) Tumour fraction (TFx) estimated from methylation signals at different treatment timepoints (baseline, C1D1, C2D1, pre‐surgery and post‐surgery) across all patients (*n* = 14). (B) Heatmap of promoter‐region differentially methylated fragments (DMFs) at baseline. (C) Gene Ontology (GO) enrichment of hypermethylated DMFs. (D) ERBB2 copy number variation (CNV) in Pt05 showing copy number gain at baseline.

Plasma samples were longitudinally obtained from 14 patients at baseline and at each treatment cycle. Genome‐wide cfDNA methylation profiling was conducted using the PredicineEPIC assay. TFx estimated from methylation patterns showed a decreasing trend at the population level across treatment timepoints (Figure 5A). Individual patient trajectories are provided in the Supporting Information (Figure S1) to illustrate inter‐individual variability in TFx dynamics. Heatmap clustering of DMFs in promoter regions revealed distinct methylation signatures at baseline, indicating the potential utility of cfDNA methylation for early molecular classification (Figure 5B). DMFs located in promoter regions were annotated to their corresponding genes. GO enrichment analysis based on these gene annotations identified several biological processes potentially associated with tumour development and cell proliferation (Figure 5C). Furthermore, CNV analysis of ERBB2 in plasma showed a significant copy number gain (CNV > 2.2) in the baseline sample of one patient (Pt05), consistent with tissue HER2‐overexpressing (Figure 5D). The ERBB2 copy number decreased after one cycle of therapy in this patient. Given the limited sample size, these findings should be considered exploratory, and formal statistical analyses correlating ctDNA dynamics with pathological response were not performed.

### 3.6 Survival outcomes

**FIGURE 6:**
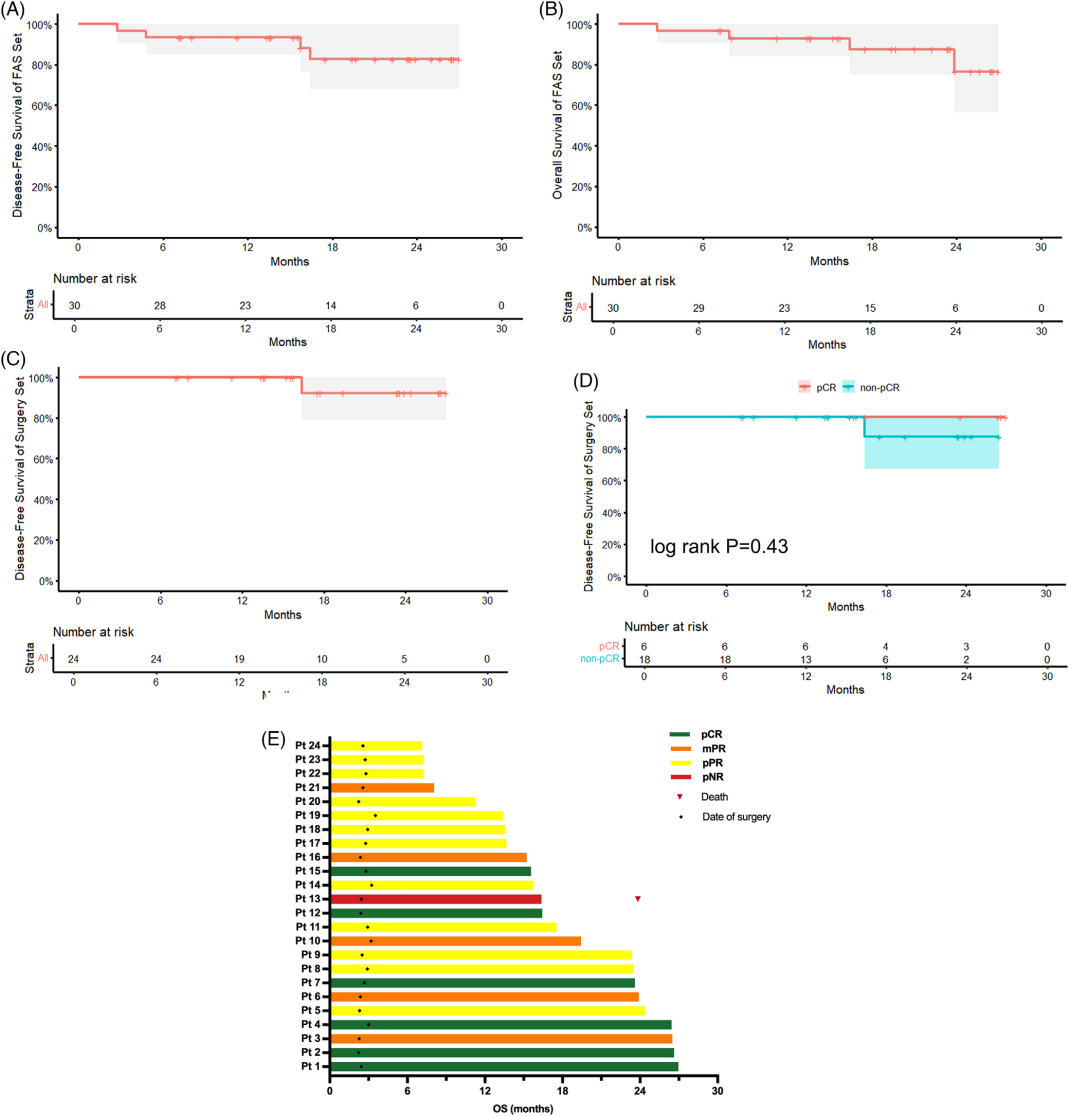
Survival outcomes of all patients. (A) Disease‐free survival of full analysis set (FAS) set (*n* = 30). (B) Overall survival of FAS set (*n* = 30). (C) Disease‐free survival of surgery set (*n* = 24). (D) Disease‐free survival of pCR and n‐pCR patients of surgery set (*n* = 24). (E) Pathological response and follow‐up of each patient were censored at the time of the procedure (*n* = 24).

At the data cutoff of 10 May 2025, median follow‐up among surviving patients was 19.4 months (95% CI: 8.0–25.6). Death occurred in four of 30 cases (13.3%) in the FAS set, while relapse was observed in one of 24 cases (4.2%) in the surgical set. In total, six cases (20.0%) had a follow‐up duration exceeding 2 years. The median DFS (95% CI: NR–NR) and median OS (95% CI: 25.9–NR) were not reached in FAS set and surgery set (95% CI: NR–NR) (Figure 6A‒C). At 18 months, DFS rates were 82.6% (95% CI: 68.1%–100.0%) in the FAS set and 87.5% (95% CI: 74.8%–100.0%) in the surgery set. The corresponding OS rate in the FAS set was 92.3% (95% CI: 78.9%–100.0%). Patients achieving pCR demonstrated a numerically higher DFS (*p* = .43) and OS compared with those without pCR (TRG 1–3; Figure 6D). The pathological response and follow‐up of each patient were assessed and shown in Figure 6E.

### 3.7 Subgroup analysis by biomarker status

In the surgical cohort, pathological TRG analysis showed that six patients (25.0%) achieved TRG1a, 11 patients (45.8%) achieved TRG1a+1b and 13 patients (54.2%) had TRG2–3 (Table 4). Gastric tumours were predominant across all TRG subgroups. Patients with TRG1a were evenly distributed between G2 and G3 histology, while most TRG2–3 cases were G2. The majority of tumours were MSS/pMMR, and MSI‐H/dMMR was rare. HER2 3+ expression and PD‐L1 CPS ≥1 were more frequent in patients with poorer pathological response (TRG2–3), suggesting potential associations between biomarker status and pathological outcomes. The therapeutic efficacy was further stratified by HER2 and PD‐L1 expression levels. In HER2 2+ and 3+ subgroups (Table 5), the ORRs were both 75%. The pCR rates were 25.0% and 6.2%, respectively (*p* = .338). A similar pattern was observed for MPR, with rates of 25.0% and 6.2%, respectively, again without statistical significance (*p* = .338). In the PD‐L1 subgroup analysis (Table 6), the ORR was higher in CPS ≥1 patients (81.8%) compared to CPS <1 patients (60.0%). Similarly, the DCR reached 100% in CPS ≥1 patients. However, pCR and MPR rates showed no significant differences between the groups, suggesting that PD‐L1 may better predict radiological than pathological response.

**TABLE 4:** Pathological response in the subgroup of surgery set.

Pathological response	TRG1a (*n* = 6)	TRG 1a+1b (*n* = 11)	TRG 2+3 (*n* = 13)
Primary tumour location, *n* (%)			
Gastric	4 (66.7%)	6 (54.5%)	9 (69.2%)
Gastroesophageal junction	2 (33.3%)	5 (45.5%)	4 (30.8%)
Histologic grade, *n* (%)			
G2	3 (50.0%)	6 (54.5%)	8 (61.5%)
G3	3 (50.0%)	4 (36.4%)	3 (23.1%)
Not reported or invalid	0 (.0%)	1(9.1%)	2 (15.4%)
MSI status, *n* (%)			
MSI‐H/dMMR	1 (16.7%)	1 (9.1%)	0 (.0%)
MSS/pMMR	4 (66.7%)	8 (72.7%)	10 (92.3%)
Not reported or invalid	1 (16.7%)	2(18.2%)	3 (23.1%)
HER2 status, *n* (%)			
2+	4 (66.7%)	7 (63.6%)	6 (46.2%)
3+	2 (33.3%)	4 (36.4%)	7 (53.8%)
PD‐L1 CPS, *n* (%)			
CPS < 1	1 (16.7%)	2 (18.2%)	3 (23.1%)
CPS ≥ 1	1 (16.7%)	4 (36.4%)	7 (53.8%)
Not reported or invalid	4 (66.7%)	5 (45.5%)	3 (23.1%)

Abbreviations: MSI, microsatellite instability; MSS/pMMR, microsatellite stability/proficient mismatch repair.

**TABLE 5:** Comparison of therapeutic efficacy between HER2 2+ and HER2 3+ subgroups.

Best responses	HER2 2+ (*n* = 16)	HER2 3+ (*n* = 16)	*p*‐Value
Complete response	2 (12.5)	1 (6.2)	
Partial response	10 (62.5)	11 (68.8)	
Stable disease	2 (12.5)	2 (12.5)	
Progressive disease	1 (6.2)	1 (6.2)	
ORR	12 (75.0)	12 (75.0)	1.000
DCR	14 (87.5)	14 (87.5)	1.000
pCR	4 (25.0)	1 (6.2)	.338
MPR	4 (25.0)	1 (6.2)	.338

Abbreviations: DCR, disease control rate; MPR, major pathological response; ORR, objective response rate; pCR, pathological complete response.

**TABLE 6:** Comparison of therapeutic efficacy between PD‐L1 CPS < 1 and PD‐L1 CPS ≥ 1 subgroups.

Best responses	PD‐L1 CPS < 1 (*n* = 10)	PD‐L1 CPS ≥ 1 (*n* = 11)
Complete response	0 (.0)	1 (9.1)
Partial response	6 (60.0)	8 (72.7)
Stable disease	1 (10.0)	2 (18.2)
Progressive disease	1 (10.0)	0 (.0)
ORR	6 (60.0)	9 (81.8)
DCR	7 (70.0)	11 (100.0)
pCR	2 (20.0)	1 (9.1)
MPR	3 (30.0)	3 (27.3)

Abbreviations: DCR, disease control rate; MPR, major pathological response; ORR, objective response rate; pCR, pathological complete response.

### 3.8 Univariate analysis for pCR and MPR predictors

To identify potential predictors for pathological response, univariate logistic regression was conducted (Tables 7 and 8). None of the tested variables, including age, primary tumour location, clinical T or N stage, and histologic grade, were significantly associated with either pCR or MPR (all *p* > .05). These findings indicate that no clinical baseline factors strongly predicted pathological efficacy in this cohort.

**TABLE 7:** Univariate logistic regression for pathological complete response (pCR).

Variable	OR	95% CI	*p*‐Value
Age	1.09	(.91, 1.30)	.3599
Primary tumour location (GC vs. GEJ)	2.40	(.26, 22.10)	.4396
Clinical T stage (cT4 vs. cT3)	3.38	(.29, 39.32)	.3316
Clinical N stage (cN2/3 vs. cN1)	.30	(.03, 3.45)	.3316
Histologic grade (G3 vs. G2)	.61	(.05, 7.24)	.6962

Abbreviations: CI, confidence interval; GC, gastric cancer; GEJ, gastroesophageal junction; OR, odds ratio.

**TABLE 8:** Univariate logistic regression for major pathological response (MPR).

Variable	OR	95% CI	*p*‐Value
Age (years)	1.08	(.91, 1.28)	.3839
Primary tumour location (GC vs. GEJ)	.82	(.17, 4.02)	.8078
Clinical T stage (cT4 vs. cT3)	1.31	(.27, 6.45)	.7379
Clinical N stage (cN2/3 vs. cN1)	.54	(.10, 2.99)	.4797
Histologic grade (G3 vs. G2)	1.07	(.18, 6.29)	.9383

Abbreviations: CI, confidence interval; GC, gastric cancer; GEJ, gastroesophageal junction; OR, odds ratio.

### 3.9 Safety profile

During the neoadjuvant period, 24 patients (75.0%) experienced treatment‐related adverse events (TRAEs, shown in the Table 9). Neutropenia (*n* = 10, 31.3%), anaemia (*n* = 8, 25.0%) and lymphopenia (*n* = 6, 18.8%) were the most frequent TRAEs, followed by elevated alanine aminotransferase (*n* = 5, 15.6%), elevated aspartate aminotransferase (*n* = 4, 12.5%), leukopenia (*n* = 3, 9.4%), vomiting (*n* = 2, 6.3%) and hyperbilirubinaemia (*n* = 2, 6.3%). Notably, 10 patients (31.3%) experienced grade ≥3 TRAEs, with neutropenia being the most frequent severe event (*n* = 3, 9.4%). Two patients experienced a treatment delay in neoadjuvant treatment; however, no patients postponed surgery due to adverse events. No clinically significant peripheral neuropathy, ocular toxicity, reactive cutaneous capillary endothelial proliferation (RCCEP) or other immune‐related adverse events were observed during the study period. Importantly, no perioperative surgical complications were observed among the 24 patients who underwent gastrectomy with D2 lymphadenectomy, further supporting the feasibility and safety of this neoadjuvant regimen.

**TABLE 9:** Treatment‐related adverse events (TRAEs) in the safety analysis set (SAS) population (*n* = 32).

	All grade, *n* (%)	Grade 3‒4, *n* (%)
All adverse events	24 (75.0%)	10 (31.3%)
Neutropenia	10 (31.3%)	3 (9.4%)
Anaemia	8 (25.0%)	1 (3.1%)
Lymphopenia	6 (18.8%)	1 (3.1%)
Increased ALT	5 (15.6%)	2 (6.3%)
Increased AST	4 (12.5%)	1 (3.1%)
Leukopenia	3 (9.4%)	1 (3.1%)
Vomiting	2 (6.3%)	1 (3.1%)
Hyperbilirubinaemia	2 (6.3%)	0
Decreased appetite	1 (3.1%)	0
Fever	1 (3.1%)	0
Hypodynamia	1 (3.1%)	0
Decreased platelet count	1 (3.1%)	0
Intestinal obstruction	1 (3.1%)	0
Hyperthyroidism	1 (3.1%)	0
Infectious pneumonia	1 (3.1%)	0
Increased CPK	1 (3.1%)	0

Abbreviations: ALT, alanine aminotransferase; AST, aspartate aminotransferase; CPK, creatine phosphokinase.

## 4 DISCUSSION

Despite surgery being the main curative option for locally advanced GC, high recurrence rates necessitate improved neoadjuvant therapies.^28^, ^29^ This study evaluates RC48 combined with camrelizumab and S‐1 as a novel neoadjuvant regimen for HER2‐overexpressing GC. In our study, 24 patients underwent D2 surgery, achieving a 100% R0 resection rate. The pCR was observed in six patients (25%), while a MPR was noted in 11 patients (45.8%), and postoperative pathological T and N staging demonstrated significant downstaging. The ORR during the neoadjuvant therapy period was 80.0% (24/30). These findings suggest encouraging antitumour activity of the RC48 combined with PD‐1 inhibitor and chemotherapy in this patient population. However, the findings should be interpreted cautiously in light of the single‐arm design. Although cross‐trial comparisons should be made carefully due to differences in study design, patient characteristics, and pathological assessment, the pathological response rates observed in our study appear numerically higher than those reported in some historical trastuzumab‐based perioperative regimens such as NEOHX, which reported a pCR rate of 9.6%. These results highlight the possible value of ADC‐based combinations in the neoadjuvant setting, which requires confirmation in larger randomised controlled trials.

With the success of anti‐HER2 therapy in advanced GC, there has been active exploration of perioperative anti‐HER2 therapy in locally advanced GC. Trastuzumab‐based combination regimens had demonstrated favourable efficacy and safety in perioperative anti‐HER2 treatment for LAGC.^30^ The NEOHX study results showed that the pCR rate was 9.6%, and the median OS was 79.9 months in HER2‐overexpressing resectable G/GEJC patients treated with trastuzumab combined with XELOX (oxaliplatin and capecitabine).^22^ The results of the Trigger and INNOVATION studies both indicated a trend towards improved pathological response rates in HER2‐overexpressing perioperative patients treated with trastuzumab combined with chemotherapy.^31^, ^32^ In our study, the regimen achieved a pCR rate of 25% and an MPR rate of 45.8%, which appears encouraging compared with previously reported perioperative regimens. Neoadjuvant regimens combining sintilimab with CapeOX and FLOT achieved pCR rates of 23.1% and 17.6%, respectively, supporting the potential benefit of incorporating ICIs into neoadjuvant therapy.^33^, ^34^ However, these studies did not specifically target the population of HER‐2 overexpression. A phase II RCT study of trastuzumab plus atezolizumab and XELOX regimen targeted HER‐2 overexpression population achieved a pCR rate of 38.1%, with a 23.8% pCR rate in the control group.^35^ Nevertheless, cross‐trial comparisons should be interpreted cautiously, as differences in study design, patient populations, treatment regimens and endpoint definitions may influence the reported outcomes. Therefore, these comparisons are intended to provide contextual reference rather than direct evidence of superiority.

ADC drugs consist of three components: monoclonal antibody, cytotoxic drug and linker. They precisely identify tumour antigens through the antibody, delivering the cytotoxic drug directly to tumour cells.^36^ Beyond direct cytotoxicity, ADCs may also modulate the tumour immune microenvironment by inducing immunogenic cell death and enhancing antitumour immune responses.^37^, ^38^ Previous preclinical studies have demonstrated that DV exhibits potent HER2‐dependent cytotoxic activity in HER2‐expressing tumour models, including GC cell lines and xenograft models, supporting the biological specificity of this HER2‐targeted ADC therapy.^39^, ^40^ These properties provide a biological rationale for combining HER2‐targeted ADCs with ICIs.

In the present study, several observations provide translational insights into this combination strategy. Patients with HER2 2+ tumours demonstrated pathological responses numerically comparable to those with HER2 3+ disease, suggesting that HER2‐targeted ADCs may retain antitumour activity even in tumours with intermediate HER2 expression. Patients with PD‐L1 CPS ≥1 also showed higher radiological responses and DCRs, consistent with findings from previous studies such as KEYNOTE‐811. These findings suggest that ADC‐mediated immune modulation may improve the efficacy of PD‐1 blockade in HER2‐overexpressing GC.

There have been no reported studies on the combination of ADCs and immunotherapy specifically for GC. The EPOC2003 study indicated that the efficacy of ADCs as single‐agent neoadjuvant therapy is limited for patients with locally advanced HER2‐overexpressing G/GEJ cancer.^21^ RC48 is the first HER2‐targeted ADC approved for marketing in China. It targets HER2‐overexpressing tumour cells and delivers the cytotoxic payload MMAE, enabling selective tumour cell killing.^41^, ^42^ Moreover, the integration of PD‐1 inhibitors and ADCs into chemotherapy significantly enhanced pathological regression. In these studies, GC patients with higher CPS scores showed better pathological responses and a better response to the combination therapy against PD‐1.^43^, ^44^ Similar predictive value of CPS was also observed in our study. Among the six patients with CPS ≥1, four patients achieved MPR. The small sample size and the proportion of patients with CPS <1 (16/32, 50.0%) limit the robustness of this finding, which should be confirmed in larger cohorts. In addition, subgroup analyses suggested that patients with HER2 2+ tumours showed comparable pathological responses to those with HER2 3+ tumours; however, no statistically significant difference was observed. This finding aligns with emerging evidence that HER2‐targeted ADCs, such as RC48, can exert antitumour effects in patients with HER2 2+ expression regardless of FISH status. Unlike traditional anti‐HER2 therapies that primarily benefit HER2 3+ or HER2 2+/FISH+ patients, ADCs have expanded the eligible treatment population to include HER2 2+ patients without gene amplification, potentially increasing the benefit‐eligible population by approximately 20%‒30%.^45^, ^46^, ^47^ This observation may reflect the complex biological behaviour of HER2 expression in GC and potential heterogeneity in HER2 immunoreactivity. Furthermore, patients with PD‐L1 CPS ≥1 demonstrated higher ORR and DCR, although pathological response differences were not statistically significant. These results support prior findings that PD‐L1 expression may be more predictive of radiological rather than pathological response in neoadjuvant settings. Nevertheless, these exploratory observations are limited by small sample size and merit further investigation. In the post hoc analysis of the study, patients with intestinal or mixed GC could benefit more from this regimen. Moreover, regardless of PD‐L1 expression or MMR status, the MPR rate in this study was significantly higher than traditional treatment regimens. Taken together, these results suggest that the immune‐modulating effects of ADCs may underlie the favourable responses observed in HER2 2+ tumours and in patients with PD‐L1 CPS ≥1 in our study, thereby supporting the clinical potential of RC48 plus PD‐1 blockade.

The exploratory cfDNA methylation analysis provided additional insight into the molecular response during neoadjuvant therapy. Methylation‐derived TFx showed a clear downward trend in most patients, reflecting the reduction of tumour burden observed radiologically and pathologically. Recent prospective studies in resectable GC have reported that ctDNA dynamics can predict pathological response and DFS.^48^, ^49^ Our data extend these findings by demonstrating that cfDNA methylation, rather than mutation‐based detection alone, can capture treatment‐related molecular changes in the perioperative setting. The analysis of methylation patterns also revealed distinct promoter‐region signatures and enriched biological processes linked to tumour proliferation and immune regulation. These observations suggest that cfDNA methylation profiling may provide an additional approach for minimal residual disease detection and early relapse monitoring, although further validation in larger prospective studies is required.^50^, ^51^ Plasma ERBB2 copy number gain detected in one patient corresponded to tissue HER2‐overexpressing, echoing evidence that cfDNA‐based HER2 CNV assessment can serve as a reliable indicator of HER2 status and therapeutic response. Such molecular concordance highlights the feasibility of using liquid biopsy to complement tissue testing, especially for real‐time monitoring of tumour evolution. Altogether, these exploratory results suggest that cfDNA methylation and CNV profiling can provide a comprehensive molecular readout of therapeutic efficacy in HER2‐overexpressing GC. Incorporating these liquid biopsy markers into future prospective studies may help refine patient selection, improve early assessment of response, and guide individualised perioperative management.^52^, ^53^

We noted a transient increase in CEA and CA19‐9 between C1D1 and C2D1 in some patients. However, tumour marker levels generally declined during the overall perioperative period. Given the limited sample size, these fluctuations should be interpreted cautiously. Further validation in larger cohorts is required.

In some neoadjuvant immunotherapy studies, pCR and MPR have shown associations with long‐term survival, although their ability to predict OS remains unclear.^54^ Therefore, the extent to which the therapeutic effects associated with pathological responses translate into long‐term survival remains to be further investigated. Given the relatively short follow‐up period in the present study (median 19.4 months), the current analysis is unable to evaluate the relationship between pathological response and long‐term survival outcomes.

Univariate logistic regression analysis failed to identify any significant baseline predictors of pCR or MPR. This suggests that traditional clinicopathologic variables, including tumour location, T/N staging and histologic grade, may be insufficient to stratify patients likely to respond to this novel combination therapy. The lack of predictive associations could be attributed to the limited sample size, inherent heterogeneity of GC, or the overriding influence of molecular factors not captured in this analysis. These findings underscore the urgent need for biomarker‐driven stratification in future trials.

In this study, the completion rate of preoperative treatment was relatively high (30/32) due to the patients' compliance. In terms of safety, this study demonstrated favourable outcomes regarding perioperative complications, mortality and the incidence of treatment‐related grade 3‒4 adverse events, with favourable patient tolerance and a manageable safety profile. The main adverse events included elevated transaminase levels and decreased neutrophil counts. No clinically significant peripheral neuropathy, ocular toxicity, RCCEP or other immune‐related adverse events were observed during the study period, and no patients required delayed surgery.

This study has several limitations. There was lack of available prior data for RC48 plus anti‐PD‐1 and chemotherapy in G/GEJ cancer patients when we designed, the sample size was empirically estimated. The relatively small sample size may result in wide CIs for efficacy estimates and limit the statistical power of subgroup analyses. Generalisability may be limited due to the single‐centre setting and the relatively small cohort size. Potential selection bias was unavoidable because lack of control group. Moreover, some patients did not proceed to surgery after neoadjuvant therapy, which may introduce additional bias when interpreting pathological response outcomes. The study primarily focused on the neoadjuvant outcomes and there was insufficient attention given to the adjuvant therapy, which could impact subsequent DFS and OS, although follow‐up was ongoing. Furthermore, the current follow‐up duration is relatively short, and survival data remain immature. Therefore, the long‐term survival benefit of this regimen requires further observation. Biomarker‐related analyses were exploratory; thus, the ability to identify new biological processes associated with treatment response is limited. In particular, the exploratory ctDNA methylation analysis was conducted in a relatively small subset of patients (*n* = 14), which limits the robustness and generalisability of these findings. Therefore, the ctDNA‐related observations should be interpreted cautiously and require validation in larger prospective studies. Further investigation integrating biomarker and translational analyses is needed to better characterise the clinical responses observed. To address these limitations, a prospective, multicentre controlled trial is planned, and larger randomised studies will be required to confirm the clinical benefit of this regimen.

## 5 CONCLUSIONS

This phase II study demonstrates that neoadjuvant therapy with RC48, camrelizumab and S‐1 is both effective and well‐tolerated in patients with resectable, HER2‐overexpressing (HER2 2+/3+, regardless of FISH) G/GEJ cancer. The combination regimen achieved encouraging pathological response rates, indicating the potential feasibility of integrating HER2‐targeted ADCs and immunotherapy into neoadjuvant treatment strategies for this population. However, the single‐arm design, limited sample size, and relatively short follow‐up render these findings preliminary. Larger randomised trials with extended follow‐up are needed to confirm these results and clarify their impact on long‐term survival.

## AUTHOR CONTRIBUTIONS

All authors have contributed to study concepts, study design, data acquisition, quality control of data and algorithms, data analysis and interpretation, statistical analysis and manuscript review.

## CONFLICT OF INTEREST STATEMENT

The authors declare that they have no known competing financial interests or personal relationships that could have appeared to influence the work reported in this paper.

## ETHICS STATEMENT

The study received approval from the Institutional Ethics Committee of Shandong Cancer Hospital and Institute, Shandong First Medical University (SDZLEC2022‐152‐02).

## Supporting information




**FIGURE S1**. Individual trajectories of circulating tumour DNA (ctDNA) tumour fraction during neoadjuvant therapy. Longitudinal changes in tumour fraction (TFx) estimated from methylation signals are shown for each individual patient (*n* = 14) across treatment timepoints (baseline, C1D1, C2D1, pre‐surgery and post‐surgery). Each panel represents a single patient, illustrating inter‐individual variability in ctDNA dynamics.

## Data Availability

Data will be made available upon request.
