# Correction to “Efficacy and safety of disitamab vedotin (RC48) combined with camrelizumab and S‐1 for neoadjuvant therapy of locally advanced gastric cancer with HER2‐overexpressing: Preliminary results of a prospective, single‐arm, phase II study”

**DOI:** 10.1002/ctm2.70775

**Published:** 2026-08-05

Wang L, Liu B, Liu L, et al. Efficacy and safety of disitamab vedotin (RC48) combined with camrelizumab and S‐1 for neoadjuvant therapy of locally advanced gastric cancer with HER2‐overexpressing: Preliminary results of a prospective, single‐arm, phase II study. *Clin Transl Med*. 2026;16:e70679. doi:10.1002/ctm2.70679

1. In Section 3.1, the sentence: “Regarding immunohistochemical baseline characteristics, 24 patients (75.0%) exhibited microsatellite stability/proficient mismatch repair (MSS/pMMR), and 15 patients (46.9%) were classified as HER2 3+.” was incorrect. This should have read: “Regarding immunohistochemical baseline characteristics, 24 patients (75.0%) exhibited microsatellite stability/proficient mismatch repair (MSS/pMMR), and 16 patients (50.0%) were classified as HER2 3+.”
2. In Table 4, the percentage for “MSS/pMMR” in the TRG2+3 subgroup was incorrectly reported as “92.3%”. The correct value is “76.9%”.
3. In Table 5:
  1. The pCR rate in the HER2 3+ subgroup was incorrectly reported as “1 (6.2%)”. The correct value is “2 (12.5%)”.
  2. The MPR rate in the HER2 2+ subgroup was incorrectly reported as “4 (25.0%)”. The correct value is “7 (43.8%)”.
  3. The MPR rate in the HER2 3+ subgroup was incorrectly reported as “1 (6.2%)”. The correct value is “4 (25.0%)”.
  4. The corresponding *p*‐values were corrected from “0.338” to “0.654” for pCR and “0.458” for MPR.
4. In Section 3.7, the sentence: “The pCR rates were 25.0% and 6.2%, respectively (*p* = .338). A similar pattern was observed for MPR, with rates of 25.0% and 6.2%, respectively, again without statistical significance (*p* = .338).” should have read: “The pCR rates were 25.0% and 12.5%, respectively (*p* = .645). A similar pattern was observed for MPR, with rates of 43.8% and 25.0%, respectively, again without statistical significance (*p* = .458).”
5. In Table 6:
  1. The pCR rate in the PD‐L1 CPS < 1 subgroup was incorrectly reported as “2 (20.0%)”. The correct value is “1 (10.0%)”.
  2. The MPR rate in the PD‐L1 CPS < 1 subgroup was incorrectly reported as “3 (30.0%)”. The correct value is “2 (20.0%)”.
  3. The MPR rate in the PD‐L1 CPS ≥ 1 subgroup was incorrectly reported as “3 (27.3%)”. The correct value is “4 (36.4%)”.
6. In Discussion, the sentence: “Among the six patients with CPS ≥ 1, four patients achieved MPR. The small sample size and the proportion of patients with CPS < 1 (16/32, 50.0%) limit the robustness of this finding, which should be confirmed in larger cohorts.” should have read: “Among the 11 patients with CPS ≥ 1, four patients achieved MPR. The small sample size and the proportion of patients with CPS ≥ 1 (11/32, 34.4%) limit the robustness of this finding, which should be confirmed in larger cohorts.”

We apologise for these errors.

