# Supplementary figures and images for: Efficacy and safety of disitamab vedotin (RC48) combined with camrelizumab and S‐1 for neoadjuvant therapy of locally advanced gastric cancer with HER2‐overexpressing: Preliminary results of a prospective, single‐arm, phase II study
Source: Clin Transl Med. 2026 May 6;16(5):e70679. doi: 10.1002/ctm2.70679 (PMC13146350; doi:10.1002/ctm2.70679)

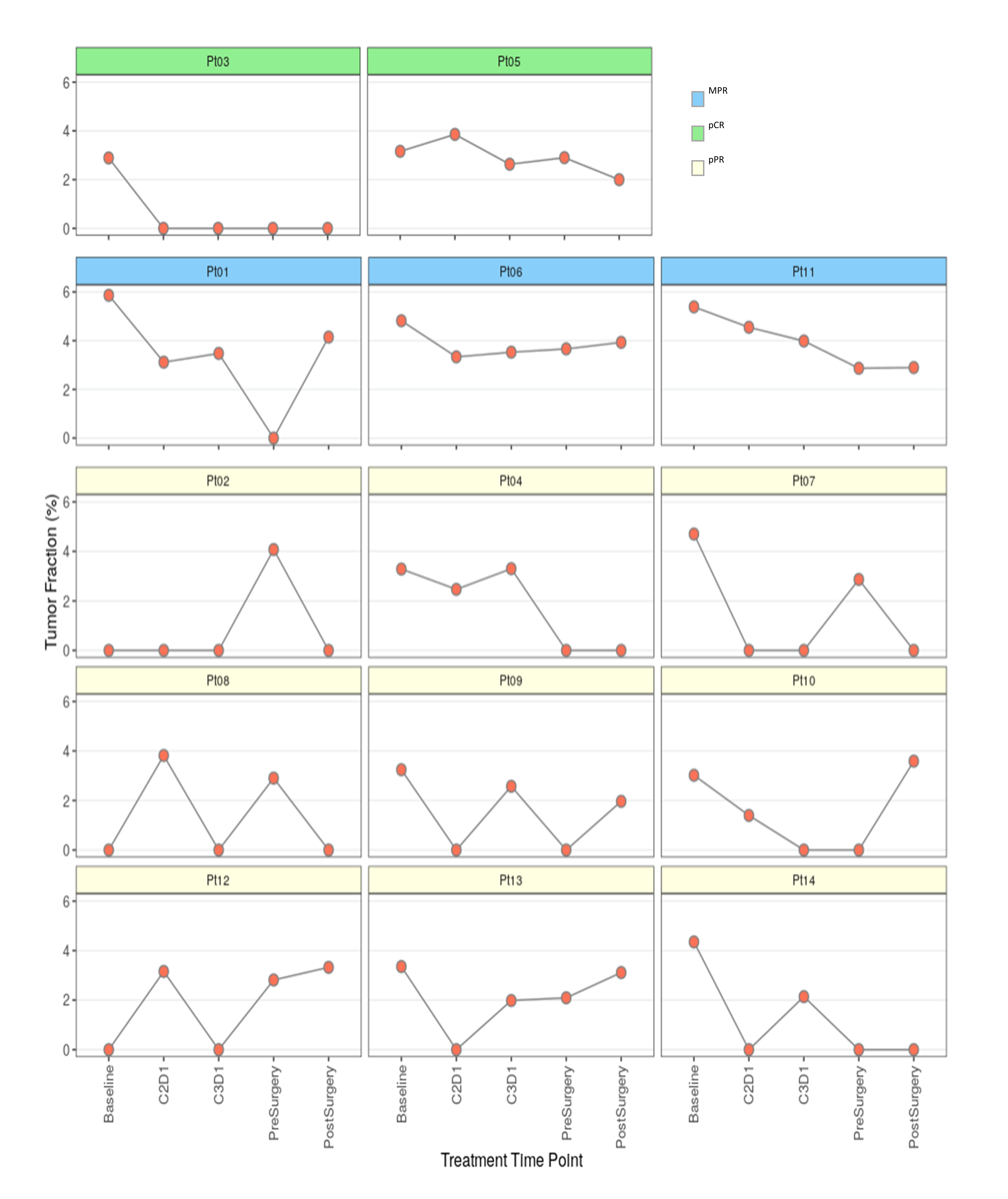

Supplement: Supplementary file 1 — FIGURE S1. Individual trajectories of circulating tumour DNA (ctDNA) tumour fraction during neoadjuvant therapy. Longitudinal changes in tumour fraction (TFx) estimated from methylation signals are shown for each individual patient (n = 14) across treatment timepoints (baseline, C1D1, C2D1, pre‐surgery and post‐surgery). Each panel represents a single patient, illustrating inter‐individual variability in ctDNA dynamics. [file CTM2-16-e70679-s001.png]
